# BACE1 Inhibition Increases Susceptibility to Oxidative Stress by Promoting Mitochondrial Damage

**DOI:** 10.3390/antiox10101539

**Published:** 2021-09-28

**Authors:** Carolina Francelin, Sayak K. Mitter, Qingwen Qian, Sandeep Kumar Barodia, Colin Ip, Xiaoping Qi, Hongmei Gu, Judith Quigley, Matthew S. Goldberg, Maria B. Grant, Michael E. Boulton

**Affiliations:** 1 Department of Ophthalmology and Visual Sciences, University of Alabama at Birmingham, Birmingham, AL 35233, USA; crovarotto@uabmc.edu (C.F.); skmitteruab@gmail.com (S.K.M.); xqi@uabmc.edu (X.Q.); mariagrant@uabmc.edu (M.B.G.); 2 Department of Ophthalmology, Indiana University School of Medicine, Indianapolis, IN 46202, USA; qingwen-qian@uiowa.edu (Q.Q.); fccsip@gmail.com (C.I.); guhongmei2000@hotmail.com (H.G.); judi.quigley13@gmail.com (J.Q.); 3 Center for Neurodegeneration and Experimental Therapeutics, Department of Neurology, University of Alabama at Birmingham, Birmingham, AL 35233, USA; sandipbarodia@gmail.com (S.K.B.); mattgoldberg@uabmc.edu (M.S.G.)

**Keywords:** oxidative stress, BACE1, mitochondrial damage, ARPE19

## Abstract

BACE1 is a key enzyme facilitating the generation of neurotoxic β-amyloid (Aβ) peptide. However, given that BACE1 has multiple substrates we explored the importance of BACE1 in the maintenance of retinal pigment epithelial (RPE) cell homeostasis under oxidative stress. Inhibition of BACE1 reduced mitochondrial membrane potential, increased mitochondrial fragmentation, and increased cleaved caspase-3 expression in cells under oxidative stress. BACE1 inhibition also resulted in significantly lower levels of mitochondrial fusion proteins OPA1 and MFN1 suggesting a higher rate of mitochondrial fission while increasing the levels of mitophagic proteins Parkin and PINK1 and autophagosome numbers. In contrast, BACE2 had minimal effect on cellular response to oxidative stress. In summary, our results emphasize the importance of BACE1 in augmenting cellular defense against oxidative stress by protecting mitochondrial dynamics.

## 1. Introduction

β-Site amyloid precursor protein cleaving enzyme (β-secretase or BACE) is strongly expressed in the brain and is widely known as one of the key enzymes in the etiology of Alzheimer’s disease (AD) [1]. BACE catalyzes the rate-limiting first step in the sequential proteolytic cleavage of amyloid precursor protein (APP) to generate Aβ, which is deposited in the amyloid plaques associated with Alzheimer’s disease (AD) [2]. There are two β-secretase enzymes, namely BACE1, which is a 501 amino acid type 1 transmembrane aspartic protease, and BACE2, which shares approximately 68% homology with BACE1 [3]. BACE1 levels are reported to be highest in the brain [1,4], while BACE2 is expressed at much lower levels and has less APP cleaving activity. Although it is widely known that BACE1 is highly expressed in the brain, there has been surprisingly limited investigation into the role of BACE in other tissues, even though BACE1 has been shown to be constitutively expressed in a range of tissues, including the pancreas, heart, and retina [5,6,7,8,9]. Furthermore, BACE1 cleaves, in addition to APP, an expanding number of substrates including neuregulin, interleukin-1 receptor 2, LDL receptor-related protein, vascular endothelial growth factor receptor-1 (VEGFR1), voltage-gated sodium (Na_v_) channel β2-subunit (Na_v_β_2_), and potassium (K_v_) channel subunits KCNE1 and KCNE2 [2,5,10].

We have previously reported that both BACE1 and BACE2 are strongly expressed in the normal retina and that BACE1^–/–^ mice develop significant retinal pathology suggestive of vascular abnormalities, greater susceptibility to oxidative stress, and lysosomal perturbation [5]. In the present study, we attempted to establish the role of BACE1 and BACE2 in protection of retinal pigment epithelial (RPE) cells against oxidative stress using an epithelial cell culture system and to examine mitochondrial characteristics following BACE inhibition. Our data demonstrate that BACE1, but not BACE2, is a key player in epithelial RPE cell homeostasis and that BACE1 inhibition impacts mitochondrial dynamics and primes the cell for initiating cell death pathways.

## 2. Materials and Methods

### 2.1. Cell Culture

Human retinal pigmented epithelial cell line ARPE19 purchased from ATCC (Manassas, VA, USA) was cultured at 37 °C and 5% CO_2_ in Ham’s F-10 medium (Corning, Glendale, AZ, USA) supplemented with 10% fetal bovine serum and antibiotics (media refreshed every 48 h). For all experimental purposes, cells were seeded (within 25 passages) at a 1:3 ratio.

### 2.2. Transient Knockdown of BACE1 and BACE2

After reaching 80% confluence, ARPE19 cells (on either 6 or 96-well plates) were transfected with validated BACE1, BACE2, or non-specific scrambled siRNAs (Cat nos. s24219, s24560, and 4390843, respectively; Ambion—Waltham, MA, USA) using Lipofectamine RNAiMax^®^ (Invitrogen, Waltham, MA, USA) in serum-free OptiMEM^®^ media.

### 2.3. Oxidative Stress Protocol

After siRNA transfection (24 h), cells were subjected to oxidative stress mediated by H_2_O_2_ (0.4 or 0.8 mM) or Rotenone (5 or 10 µM) for 4 or 24 h, as indicated in the figure legends.

### 2.4. Cytotoxicity Assay

Lactate dehydrogenase was measured using the CytoTox 96^®^ non-radioactive cytotoxicity assay kit (Promega Corporation, Madison, WI, USA). Briefly, 100 μL of the media from each condition were transferred to a 96-well plate. The remaining medium was aspirated and lysis buffer was added onto the cells. Reaction solution was mixed with both supernatant and cell lysate and incubated at room temperature for 30 min before adding stop buffer to arrest the reaction. Absorbance intensity was measured at 490 nm using a plate reader. The data were presented as the ratio to the untreated control.

### 2.5. β-Secretase Activity Assay

β-Secretase activity in ARPE19 cells subjected to oxidative stress was measured using the β-secretase activity fluorometric assay kit (Biovision, Milpitas, CA, USA). Briefly, after oxidative stress challenge, cells were collected by centrifugation following trypsinization, lysed in extraction buffer, and incubated on ice for 10 min. Equal amounts of protein (by weight) were loaded to black 96-well plates, and reaction buffer was added followed by β-secretase substrate and incubation at 37 °C for 1 h. The plate was read using a fluorescent microplate reader (Biotek ^®^, Winooski, VT, USA) (excitation/emission: 350 nm/500 nm).

### 2.6. Amyloid β 40 Assay

Cell culture-conditioned media from ARPE19 cells on T-75 flasks were collected at the end of the experimental time-course and analyzed for amyloid β 40 residue using the colorimetric FastScan^™^ β-Amyloid (1–40) ELISA Kit #20882 (Cell Signaling, Danvers, MA, USA) according to the manufacturer’s instructions. Absorbance was measured using a spectrophotometer at 450 nm.

### 2.7. DNA Fragmentation Detection

Cytoplasmic histone-associated DNA fragments were detected using the cell death ELISA kit from Roche Diagnostics (Indianopolis, IN). Briefly, after incubation with oxidative stressors, cells were lysed and centrifuged at 200 g (10 min), and immunogen and supernatant mix was applied on the ELISA well and incubated for 2 h at room temperature. After washing, substrate solution was added to the wells and incubated until color change that was measured at 405 nm. After background absorbance subtraction, DNA fragment enrichment factor of each sample was calculated as a ratio of sample absorbance to untreated control.

### 2.8. Mitochondrial Membrane Potential (Δψ_m_)

Δψ_m_ was determined by cationic fluorescent tetramethylrhodamine (TMRM) dye (Thermo Scientific, Norcross, GA, USA), which accumulates specifically in bioenergetically active mitochondria. The dye diffuses out of mitochondria that have lower membrane potential. Before treatment endpoint, ARPE19 cells were loaded with TMRM (50 nM) for 30 min at 37 °C and trypsinized, and pellets were resuspended in PBS and immediately assessed by flow cytometry (excitation/emission: 510/580 nm). Data were analyzed using FlowJo^TM^ Software for Windows Version 10, Ashland, OH, USA).

### 2.9. Mitochondrial Morphology

Mitochondria of ARPE19 cells transfected with siRNA were labeled with BacMam 2.0 CellLight^TM^ Mito-RFP (Invitrogen, Waltham, MA, USA)). Briefly, after withdrawing siRNA transfection reagent complexes, Mito-RFP was applied for 24 h at a ratio of 20 viral particles/cell based on approximately 3.5 × 10^4^ cells/well at 80% confluence. Cells were fixed with 4% paraformaldehyde on completion of experiment and analyzed by confocal microscopy. The mitochondrial network was evaluated on multiple occasions throughout the study using ImageJ (https://imagej.nih.gov/ij/) and the Mito Morphology Macro (http://imagejdocu.tudor.lu/doku.php?id=plugin:morphology:mitochondrial_morphology_macro_plug-in:start#installation), as described by Dagda et al. [10]. Briefly, images acquired of RPE transduced with Mito-RFP were pseudo-colored in green channel followed by the setting of thresholds. Mitochondrial interconnectivity index (average mitochondrial area/perimeter ratio) was normalized to circularity (to account for mitochondrial swelling) and was plotted. At least 25 cells/treatment per experiment were analyzed.

### 2.10. Mitophagy

To assess mitophagy, ARPE19 cells were transiently transfected with SB mito-QC, a plasmid construct prepared by fusing the Tom20 mitochondrial targeting sequence (consisting of the first 35 amino acids of Tom20) in frame with the coding sequence of EGFP and mCherry in tandem. After the indicated treatments, transfected cells were fixed in 4% PFA, and confocal images were acquired using 63 × oil-immersion objective. Red and yellow puncta were quantified by manual counting. The SB-Mito-QC reporter localizes to mitochondria and appears yellow (red + green), while mitochondria undergoing autophagy and delivered to lysosomes appear as red punctate structures because the EGFP signal is quenched in the acidic lysosomal pH environment. As another measure of mitophagy, ARPE19 cells were transected with GFP-Parkin to monitor the translocation of Parkin from the cytosol to the outer membrane of depolarized mitochondria (counterstained with Anti-CoxIV) using CCCP (10 µM, 4 h) as a positive control and BACE1 knockdown with or without Rotenone treatment (5 and 10 µM, 4 h). Approximately 15–30 cells in each treatment were imaged and analyzed by Mander’s overlap co-efficient within ImageJ to assess GFP-Parkin translocation to mitochondria labeled with anti-CoxIV. Rabbit anti-LC3 (Novus Biologicals, Centennial, CO, USA) followed by Alexa488^®^-conjugated anti-rabbit incubation were used to detect autophagosomes in ARPE19 cells. Autophagosome counting was performed in at least 24 cells/experiment with up to 4 experiments/group, both automatically through the Image-J “Particle Counting” plug-in and manually to confirm the results. One-way ANOVA followed by Bonferroni’s multiple comparisons test was used to calculate statistical significance, and results are shown as mean ± SEM.

### 2.11. Mitochondria Isolation

Mitochondria were isolated from confluent ARPE19 in 6 × T-150 flasks using the Qproteome kit (Qiagen, Germantown, MD, USA). Briefly, the cell pellet was lysed sequentially with lysis buffer and disruption buffers after washing in saline buffer and centrifuged at 1000× *g* to remove cell debris and then at 6000× *g* to obtain the mitochondrial pellet. The crude pellet was further purified by density gradient-based centrifugation with the supplied mitochondria purification buffer.

### 2.12. Western Blot

Cells were lysed with RIPA buffer (Sigma Aldrich, Burlington, MA, USA) supplemented with protease inhibitor cocktail (Roche), and protein concentration was measured using the BCA protein assay. Protein lysates were separated by 4–15% SDS-PAGE (Bio-Rad, Hercules, CA, USA) and transferred to PVDF membranes (0.22 µM pore size) and immunoblotted with respective primary antibodies at 4 °C overnight. Drp-1 (Cat# NB110-55288SS), phosphorylated Drp-1 (Cat# PAS-37534), caspase 3 (Cat# 9962), BAX (Cat# 5923), Lamp1 (Cat# 3293), Lamp2 (Cat# 49067), MFN-1 (Cat #14739S), MFN-2 (Cat# 11925S) and OPA1 (Cat# 80471S) antibodies were from Cell Signaling (Danvers, MA, USA). BACE1 (Cat# sc-10748) and BACE2 (Cat# sc-271212) antibodies were obtained from Santa Cruz Biotechnology, Dallas, TX, USA. PINK (Cat# BC100494) was from Novus Biologicals, Centennial, CO, USA; and Parkin (Cat# R113100) was from Biosensis, Thebarton, SA, Australia. Secondary antibodies conjugated with IRDye700DX^®^ or IRDye800^®^ (Rockland, Limerick, PA, USA) or horse-radish peroxidase (HRP) (Sigma Aldrich, Burlington, MA, USA) were incubated at room temperature for 45 min. Proteins were visualized using a c600^®^ imager (Azure Biosystems, Dublin, CA, USA) either in the IR mode (for IRDye700DX^®^ and IRDye800^®^) or in the ECL mode using ECL Prime Western Blotting Detection Reagent (GE Healthcare, Boston, MA, USA) for HRP. The relative amount of protein was calculated by densitometric analysis of bands and normalized to β-actin or GAPDH signals using ImageJ software (https://imagej.nih.gov/ij/).

### 2.13. Immunocytochemistry

To detect BACE1 colocalization with mitochondria or lysosomes, confluent ARPE19 monolayers were fixed in 4% paraformaldehyde for 10 min, washed with PBS, permeabilized, and blocked with 1% bovine serum albumin (BSA) and 5% goat serum in PBS containing 0.2% Triton X-100 for 1 h at room temperature for mitochondria colocalization and blocked with 1% BSA and 5% goat serum in PBS for lysosomal colocalization. For lysosomal staining, cells were incubated with LysoTracker^®^ Red DND-99 for 8 min prior to fixation. Following blocking, cells were incubated overnight with anti-BACE1 (Cat# sc-10748) alone on lysotracker-stained cells or with both anti-BACE1 and anti-E1 pyruvate dehydrogenase (mitochondrial marker). Following primary incubation, cells were washed and incubated with Alexa488^®^-conjugated anti-rabbit and Alexa594^®^-conjugated anti-mouse fluorescent secondary antibodies. Nuclei were counterstained with DAPI mounting medium (Vector Labs, Burkingame, CA, USA) and analyzed by confocal microscopy.

### 2.14. Statistical Analysis

Results were analyzed for statistical significance by GraphPad Prism^®^ software using ANOVA test with Tukey post hoc test (or Bonferroni test) or Mann–Whitney for comparisons wherever appropriate. Results are shown as mean ± SEM, and differences between means were considered statistically significant when *p* < 0.05.

### 2.15. Data Resources

The full Western plots used in these studies are provided as Supplementary Figure S1.

## 3. Results

### 3.1. Reduction of BACE1, but Not BACE2, Increases Susceptibility to Oxidative Stress

We have previously reported that both BACE1 and BACE2 are expressed in the RPE and that BACE1^–/–^ mice showed significant RPE pathology, including thinning, atrophy, and an increased accumulation of the intracellular age pigment, lipofuscin [5]. Interestingly, while BACE2 expression is higher in the RPE/choroid compared to BACE1, BACE2^–/–^ knockout mice showed minimal RPE pathology [5]. In this study, we sought to make a comparative evaluation of the importance of homologs BACE1 and BACE2 in maintaining RPE cell homeostasis.

**Figure 1:**
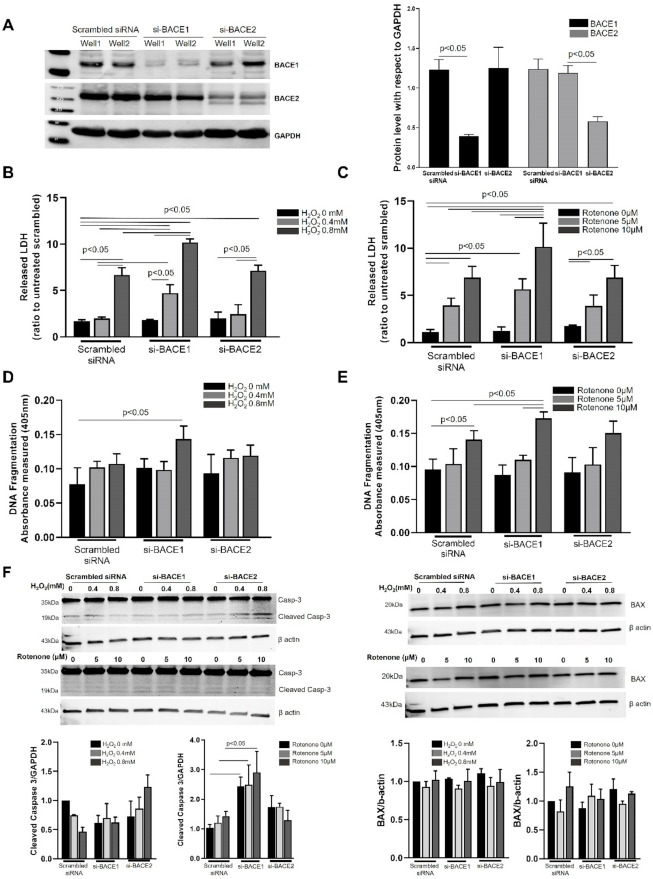
Inhibition of BACE1 but not BACE2 increased cellular susceptibility to oxidative stress. (**A**) ARPE19 cells were transfected with scrambled non-specific siRNA or siRNAs targeted specifically towards BACE1 or BACE2 for 24 h and the protein expression was analyzed by Western blotting. The bands represent mature and immature/pro-BACE1 protein. Densitometric analysis of BACE protein expression was plotted (right graph) using GAPDH as an internal control. LDH release in the cell culture media by ARPE19 cells transfected with either scrambled, BACE1 or BACE2 siRNAs in response to H_2_O_2_ (0.4 or 0.8 mM) (**B**) or Rotenone (5 or 10 µM) (**C**) was assayed 24 h after oxidative challenge using CytoTox 96^®^ non-radioactive cytotoxicity assay kit. DNA damage [14] was quantified using a cell death ELISA kit in cells transfected with either scrambled, BACE1 or BACE2 siRNAs in response to H_2_O_2_ (0.4 or 0.8 mM) (**D**) or Rotenone (5 or 10 µM) (**E**) 24 h after oxidative challenge. (**F**) 24 h after BACE1 or BACE2 knockdown, cells were subjected to Rotenone (5 or 10 µM) or H_2_O_2_ (0.4 or 0.8 mM) treatment for an additional 4 h and cell lysates analyzed by Western blotting for caspase 3 and BAX. Cleaved caspase 3 is estimated with respect to GAPDH as internal control. Mean of 3 experiments was plotted with SEMs. Statistical significance was calculated by ANOVA or Mann–Whitney tests and differences were considered significant when *p* < 0.05.

We first investigated the role of BACE1 and BACE2 in regulating RPE cell survival against extracellular H_2_O_2_-induced oxidative stress and intracellular mitochondrial stress induced by the mitochondrial complex I inhibitor, Rotenone [11]. Transient siRNA targeting of either BACE1 or BACE2 showed robust knockdown of their respective gene targets (83 and 82% respectively) up to 48 h after transfection compared to scrambled siRNA in RPE cells confirmed by Western blot (Figure 1A). Twenty-four hours after knockdown of either BACE1 or BACE2, we subjected the cells to H_2_O_2_ (0.4 and 0.8 mM)- and Rotenone (5 and 10 µM)-mediated oxidative stress and assayed for cell survival 24 h after challenge. Lactate dehydrogenase (LDH) release is a characteristic feature of late stage apoptosis and early necrosis during cellular demise [12]. Twenty-four hours after treatment with 0.8 mM H_2_O_2_, untransfected or scrambled siRNA transfected RPE cells showed an almost 7-fold increase in LDH release in the medium compared to the respective untreated scrambled control (Figure 1B). The lower 0.4 mM H_2_O_2_ concentration did not show a significant increase in LDH release. However, both concentrations of H_2_O_2_ showed a dramatic increase in LDH release of 4- and 1.5-fold in BACE1, but not BACE2 knockdown cells, at 0.4 mM and 0.8 mM H_2_O_2_, respectively, compared to cells receiving scrambled siRNA (Figure 1B). Similarly, 24 h after treatment with Rotenone, cells showed increased LDH release at both 5 and 10 µM Rotenone (4- and 7-fold, respectively) compared to scrambled control. BACE1 knockdown resulted in a small but significant increase of 1.2-fold in 10 µM Rotenone-treated BACE1 knockdown cells compared to scrambled cells receiving 10 µM Rotenone. Neither 5 µM Rotenone nor BACE2 at either Rotenone concentration resulted in any difference to LDH in cells receiving Rotenone + scrambled siRNA (Figure 1C).

Oxidation-induced apoptosis of RPE cells has been widely reported and is characterized by DNA fragmentation [13]. We observed a significant increase in DNA fragmentation in scrambled siRNA transfected cells exposed to 10µM Rotenone, but not H_2_O_2_, and this was further increased in BACE1 knockdown RPE cells during 10 µM Rotenone and 0.8 mM H_2_O_2_ oxidative stress (Figure 1D,E). To assess if there was induction of apoptotic machinery upon BACE inhibition, we evaluated the levels of apoptotic effector molecules Caspase 3 and BAX, a pro-apoptotic member of the Bcl-2 protein family, in the presence or absence of H_2_O_2_ or Rotenone. Pro-caspase 3 (molecular weight 35 KDa) displayed a prominent cleaved form (molecular weight 19 KDa), suggesting that BACE inhibition alone is sufficient to initiate the apoptotic mechanism (Figure 1F). While oxidative stress with Rotenone (10 µM) at 24 h resulted in increased cleaved caspase 3 in scrambled siRNA transfected cells, BACE1 knockdown cells showed a significantly higher caspase-3 cleaved form in matched treatments compared to scrambled control. BACE2 knockdown cells, however, did not show a higher level of apoptotic induction than scrambled siRNA transfected cells. However, neither BACE1 or BACE2 knockdown resulted in any alteration in BAX levels in RPE cells compared to scrambled siRNA-treated cells for both Rotenone and H_2_O_2_ oxidative stress treatment (Figure 1F).

### 3.2. Effect of Oxidative Stress on BACE Expression and Aβ Secretion

**Figure 2:**
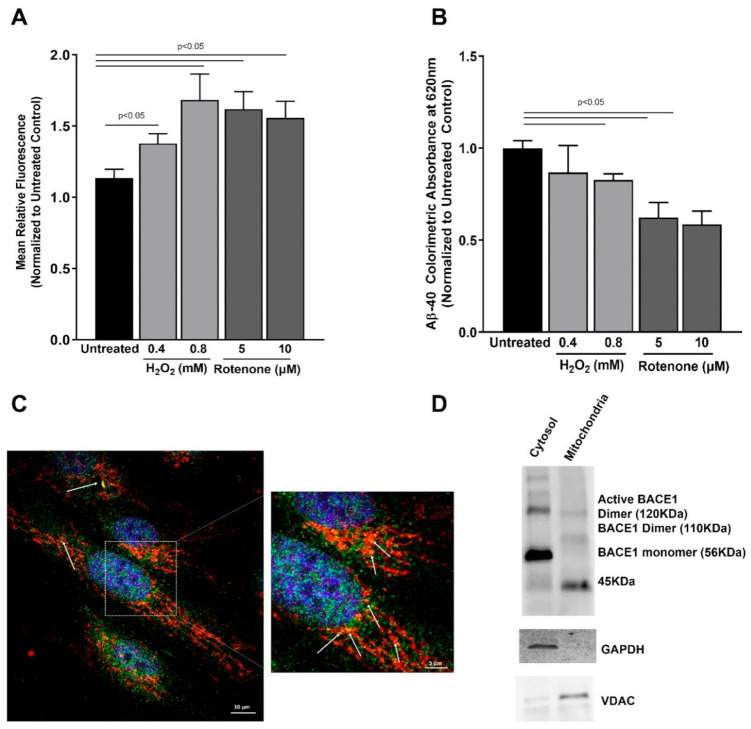
BACE1 activity, Aß-40 secretion, and RPE-mitochondria association: (**A**) 24 h after oxidative stress challenge with H_2_O_2_ (0.4 or 0.8 mM) or Rotenone (5 or 10 µM), cell lysates were analyzed Figure 1. enzyme activity; (**B**) 24 h after oxidative stress challenge with H_2_O_2_ (0.4 or 0.8 mM) or Rotenone (5 or 10 µM), cell-culture conditioned media was analyzed for amyloid β-40 residue by ELISA. (**C**) Confluent monolayers of ARPE19 stained with anti-BACE1 (green) and anti-α-subunit of E1 pyruvate dehydrogenase (red) show colocalization (white arrows). Scale bar: 10 µM. A higher magnification is shown to better visualize the colocalization. (**D**) Mitochondrial fraction was enriched from ARPE19 lysates and probed for BACE1 by Western blotting. GAPDH and VDAC were used as cytosol and mitochondria fraction enrichment controls. Data are plotted as mean ± SEM from at least 3 independent experiments. Mean differences were considered to be statistically significant when *p* < 0.05 as measured by ANOVA.

Our observations suggesting a cytoprotective role of BACE1 in RPE cells led us to ask the question whether BACE1 activity is itself increased under acute oxidative stress. Several reports have indicated an increase in BACE1 expression and activity under oxidative stress in neuronal cells, and increased BACE1 expression correlates with oxidative lipid modifications in Alzheimer’s disease patient tissue [15,16]. We observed a small but significant increase in BACE1 activity in ARPE19 cells challenged by H_2_O_2_ or Rotenone over a period of 24 h (Figure 2A). However, this increase did not correlate with an increase in secreted amyloid β-40 in the ARPE19 cell-culture media (Figure 2B). We concluded that increased BACE1 activity contributes to its cytoprotective role in the RPE under transient oxidative stress.

### 3.3. Reduction in BACE1 Expression Results in Loss of Mitochondrial Membrane Potential

Mitochondria play a critical role in cellular homeostasis. Since we observed an upregulation of apoptotic machinery in BACE1 knockdown cells subjected to oxidative stress, we evaluated the role of BACE homologs in mitochondrial homeostasis. Previous reports have indicated the presence of γ-secretase in mitochondria that plays a significant role in processing mitochondrial amyloid precursor protein [17,18]. Our observations of BACE1-mediated protection of mitochondrial homeostasis prompted us to investigate whether BACE1 is associated with mitochondria. We observed that the majority of cells in our cultures exhibited co-localization of BACE1 with mitochondria (Figure 2C), suggesting that BACE1 may play a key role in maintaining RPE mitochondrial function by direct association. This hypothesis was further strengthened by the evidence of active dimers of BACE1 (~110–120 KDa) in the enriched mitochondrial fraction of ARPE19 cell lysates (Figure 2D). We also observed a strong band (~45 KDa) in the enriched mitochondria fraction that suggests an association of BACE1 prozymogen with the RPE mitochondria [19].

**Figure 3:**
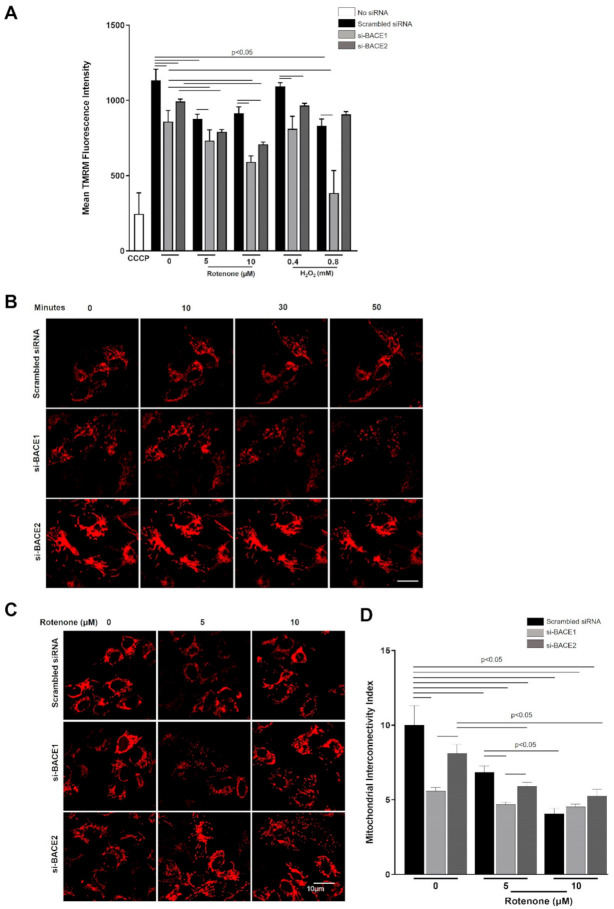
Knockdown of BACE1 expression resulted in loss of mitochondrial membrane potential and increased mitochondrial network breakdown. (**A**) Twenty-four hours after siRNA-mediated knockdown of BACE1 or BACE2, ARPE19 cells on 96-well black plates were subjected to oxidative stress with Rotenone (5 or 10 µM) or H_2_O_2_ (0.4 or 0.8 mM) for 4 h and loaded with TMRM 30 min before the end of exposure. Carbon cyanide m-chlorophenyl hydrazone (CCCP) (10 µM, 10 min) was used a positive control for mitochondrial uncoupling and membrane depolarization. (**B**) Twenty-four hours after siRNA mediated knockdown of BACE1 or BACE2, and transduction with fluorescent tagged mitochondrial vector (BacMam 2.0 CellLight^TM^ Mito-RFP labelled mitochondria), cells were subjected to oxidative stress with H_2_O_2_ (0.8 mM) and viewed by confocal microscopy at 0, 10, 30, and 50 min after challenge or rotenone (5 or 10 µM) (**C**) for 4 h and viewed by confocal microscopy. (**D**) Mitochondrial interconnectivity indices for respective treatments were plotted as mean ± SEM. Statistical significance was calculated by ANOVA, and differences in means were considered statistically significant when *p* < 0.05.

We assessed mitochondrial membrane potential using tetramethylrhodamine (TMRM) staining after subjecting cells to Rotenone or H_2_O_2_ for 4 h. As expected, the mitochondrial membrane potential (Δψ_m_) was reduced in a dose-dependent manner for each stressor (Figure 3A). Rotenone treatment resulted in a similar enhanced loss of Δψ_m_ in cells when BACE1 was knocked down under 5 µM treatment, and when BACE1 or BACE2 were knocked down and received 10 µM Rotenone, compared to the corresponding scrambled siRNA transfected control (Figure 3A). While BACE1 knockdown significantly reduced Δψ_m_ in cells exposed to both concentrations of either H_2_O_2_ or Rotenone, BACE2 inhibition resulted in Δψ_m_ reduction only for 10 µM Rotenone and 0.4 mM H_2_O_2_. We conclude that RPE mitochondrial membrane homeostasis in response to oxidative stress is predominantly dependent on BACE1 but to a lesser extent on BACE2.

### 3.4. Reduction in BACE1 Expression Enhances the Induction of Mitochondrial Fission in Cells under Oxidative Stress

Mitochondrial network dynamics (i.e., fusion and fission) are critical features of cellular homeostasis and function. Typically, higher rates of mitochondrial fission and attenuation of mitochondrial reticular structure is associated with loss of Δψ_m_ and greater susceptibility to cell death [20,21]. We hypothesized that the greater loss of Δψ_m_ when BACE1 expression was attenuated could be associated with increased mitochondrial fission. We therefore knocked down BACE1 or BACE2 in cells and subjected them to oxidative stress with H_2_O_2_ (0.8 mM) and observed Mito-RFP labelled mitochondrial morphology in live cells at 0,10, 30, and 50 min after treatment. We observed that mitochondria in scrambled siRNA transfected cells exhibited a typical reticular mitochondria morphology over this time-course, confirming healthy mitochondrial dynamics (Figure 3B). However, BACE1 knockdown resulted in dramatic fragmentation of the mitochondrial network with punctate structures appearing within 30 min post challenge (Figure 3B). BACE2 knockdown did not result in such changes in mitochondrial morphology, and reticular structures resembling those in scrambled siRNA transfected cells were maintained throughout (Figure 3B). We conclude that BACE1 activity is important for mitochondrial fission. As anticipated, Rotenone treatment for 4 h resulted in significant fragmentation of mitochondria, even in scrambled siRNA transfected cells at both 5 and 10 µM concentrations (Figure 3C). BACE1 knockdown resulted in dramatically higher mitochondrial fragmentation at either Rotenone concentration (Figure 3D), further indicating that BACE1 favors mitochondrial fusion. BACE2 knockdown did not show a significantly higher level of mitochondrial fragmentation than the corresponding scrambled siRNA transfected cells (Figure 3D).

We next investigated the key effector proteins of mitochondrial fusion and fission by Western blot. Optic atrophy 1 (OPA1), in cooperation with mitofusins Mfn1 and 2, promote mitochondrial fusion, while dynamin-related protein 1 (Drp1) is a key effector of mitochondrial fission [22]. We determined the levels of OPA1, MFNs 1 and 2, phosphorylated DRP1 (Ser637), and DRP1 in the cell lysates from cells that had been exposed to H_2_O_2_ or Rotenone for 4 h_._ Dephosphorylation of Ser637 of DRP1 is a crucial step in its activation and translocation to the mitochondrial outer membrane.

**Figure 4:**
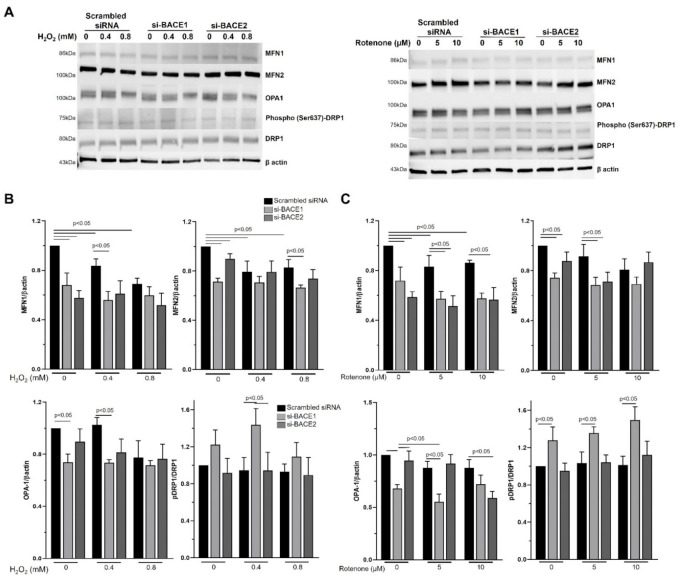
Inhibition of BACE1 expression enhanced the induction of mitochondrial fission machinery in RPE under oxidative stress. (**A**) Twenty-four hours after siRNA-mediated knockdown of BACE1 or BACE2, ARPE19 cells were subjected to oxidative stress with H_2_O_2_ (0.4 or 0.8 mM) (**A**,**B**) or Rotenone (5 or 10 µM) (**A**,**C**) for 4 h, and cell lysates were analyzed for DRP1, phospho-DRP1 (Ser637), MFN1, MFN2, and OPA1 levels by Western blotting. (**A**) A representative cropped blot from 3 experiments are shown. (**B**,**C**) Densitometric analyses of respective protein expression levels with respect to β-actin were plotted as mean ± SEM. Mean differences were considered to be statistically significant when *p* < 0.05, as measured by Mann–Whitney test.

Significantly lower levels of MFN1, MFN2, and Opa1 were observed following BACE1 knockdown compared to untreated controls in the absence of oxidative stress (Figure 4). Interestingly, BACE2 knockdown led to a reduction in MFN1 and 2 (Figure 4A). BACE1 but not BACE2 inhibition had a significant effect on the phospho-DRP1 (Ser637)/DRP1 ratio compared to untreated controls in the absence or the presence (0.4 mM H_2_O_2_ and 5 or 10 µm Rotenone) of oxidative stress (Figure 4). Oxidative stress, either H_2_O_2_ or Rotenone, had no significant effect on the expression of Opa1 or the phospho-DRP1 (Ser637)/DRP1 ratio compared to untreated controls (Figure 4B,C). However, a significant reduction in MFN1 expression was observed in cells treated with H_2_O_2_. Exposure to oxidative stress resulted in significant additional decreases in MFN1 and OPA-1 expression in BACE1 knockdown cells exposed to H_2_O_2_ and Rotenone, and OPA1 and MFN-1 expression in BACE2 knockdown cells exposed to Rotenone (Figure 4B,C). Both H_2_O_2_ (0.4 mM) and Rotenone (5 µM) increased the phospho-DRP1 (Ser637)/DRP1 ratio in BACE1 knockdown cells, which was largely due to an increase in pDRP1 and a decrease in DRP1 expression. Taken together, these results suggest that BACE1 contributes to maintaining mitochondrial fusion, and BACE1 inhibition promotes fission.

### 3.5. Mitophagic Machinery Is Upregulated in BACE1 Knockdown Cells under Oxidative Stress

**Figure 5:**
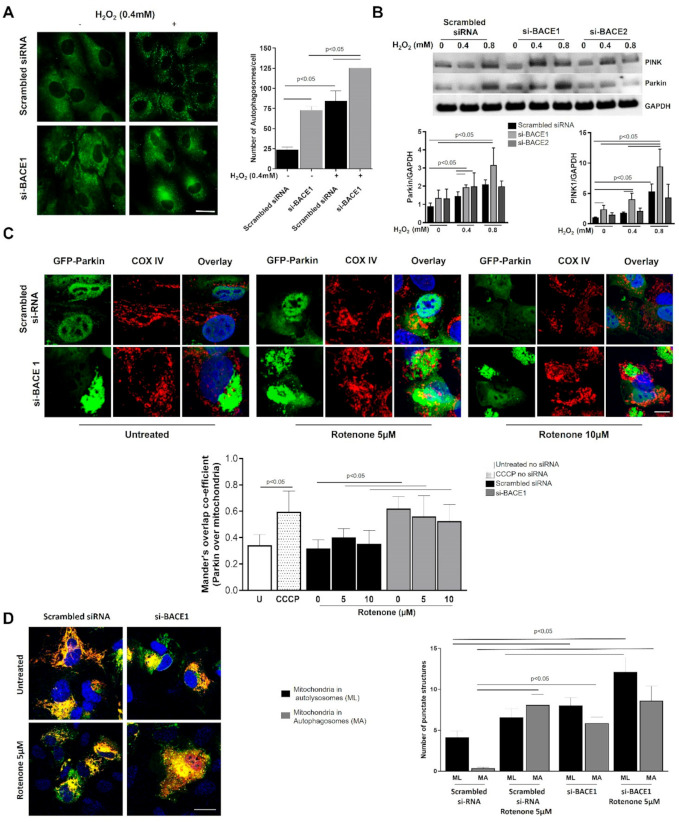
Autophagy and mitophagic machinery is upregulated in BACE1 attenuated RPE under oxidative stress. (**A**) Twenty-four after BACE1 knockdown, cells were subjected to oxidative challenge (H_2_O_2_, 0.4 mM for 4 h) followed by fixation and immunocytochemistry for LC3 to label the autophagosomes. Left panel shows representative pictures of ARPE19 cells with autophagosomes (green punctate dots). At least 24 cells/treatment/experiment were counted to plot the number of autophagosomes (right plot). (**B**) Twenty-four hours after siRNA-mediated knockdown of BACE1 or BACE2, cells were subjected to oxidative stress with H_2_O_2_ (0.4 or 0.8 mM) for 4 h, and cell lysates were analyzed for PINK1 and Parkin levels by Western blotting. Representative blots from 3 experiments are shown. Densitometric plots are shown after analyzing PINK1 and Parkin protein levels with GAPDH as internal control. (**C**) Photomicrographs of ARPE19 cells in which GFP-Parkin (green) was overexpressed, and cells were transfected with either siRNA specific to Bace1 or non-specific scrambled siRNA. Cells were treated with rotenone (5 or 10 µM), fixed and probed with anti-Cox IV (red), and nucleus counterstained with DAPI (blue). Approximately 15–30 cells in each treatment were imaged and analyzed by Mander’s overlap co-efficient within ImageJ to assess GFP-Parkin translocation to mitochondria labeled with anti-CoxIV, as shown in the graph below the images. (**D**) SB-mito-QC vector was overexpressed in ARPE19 cells transfected with either siRNA specific to Bace1 or non-specific scrambled siRNA and fixed. Images are presented of cells treated with 5 µM showing yellow (MA: mitochondria in autophagosomes) and red punctate dots (ML: mitochondria in autolysosomes). These were counted from confocal images, and data are shown graphically. Mean differences were considered to be statistically significant when *p* < 0.05, as measured by Mann–Whitney test.

To study the effect of BACE1 on RPE under oxidative stress, we investigated the role of BACE1 on RPE autophagy. Knocking down BACE1 expression itself caused a robust (up to 3-fold) increase in the number of RPE autophagosomes compared to ARPE19 cells transfected with scrambled siRNA control (Figure 5A). Oxidative stress (H_2_O_2_ 0.4 mM for 4 h) resulted in a significant (up to 3-fold) scrambled siRNA, and this was increased up to a further 1.5-fold in BACE1 knockdown cells (Figure 5A).

Parkin and PTEN-induced kinase 1 (PINK1) are key mitochondrial quality control proteins that selectively target dysfunctional mitochondria for degradation by the autophagy/lysosome system, a process referred to as mitophagy [23]. PINK1-Parkin mediated mitophagy is believed to promote cell survival by removing dysfunctional mitochondria that produce excess reactive oxygen species via leakage from the electron transport chain and by removing mitochondria that might otherwise signal apoptosis [24]. To determine if BACE knockdown affects mitophagy, we subjected the cells to oxidative stress after transiently knocking down either BACE1 or BACE2. We observed a robust dose-dependent increase in both PINK1 and Parkin levels when cells were oxidatively stressed with H_2_O_2_. BACE1 knockdown resulted in significantly increased H_2_O_2_-induced expression of both PINK1 and PARKIN compared to the scrambled siRNA transfected cells (Figure 5B), suggesting that BACE1 is protective to the mitochondria and its loss of function primes the mitophagic pathway. By contrast, compared to scrambled siRNA, no statistically significant differences in the H_2_O_2_ induced upregulation of PINK1 and Parkin were observed when BACE2 was knocked down, underlining once again the importance of the BACE1 homolog over BACE2 in cellular homeostasis.

Parkin normally localizes diffusely throughout the cytosol, but translocation of Parkin to the mitochondrial outer membrane is widely used as a functional marker of mitophagy, which is readily observed upon treatment of cells with CCCP to depolarize mitochondria [23]. Similar to CCCP treatment, si-BACE1 knockdown induced mitochondrial co-localization of transiently overexpressed GFP-Parkin both in untreated as well as Rotenone (5 and 10 µM, 4 h)-treated ARPE19 cells, supporting our hypothesis that mitophagy is primed when BACE1 is inhibited (Figure 5C).

As an independent measure of mitophagy, we generated a fluorescent mitophagy reporter (SB-Mito-QC) consisting of the N-terminal mitochondrial targeting sequence of Tom20 fused in tandem with EGFP and mCherry, similar to that previously developed and validated by others [25,26]. The red and green fluorescent signal co-localizes with mitochondria; however, any mitochondria that complete the process of mitophagy appear red because the EGFP (but not the mCherry) fluorescence is quenched upon the fusion of autophagosomes with lysosomes, due to the lower lysosomal pH. ARPE19 cells co-transfected with SB-Mito-QC and either siRNA specific for BACE1 or non-specific scrambled siRNA were treated with 5 µM Rotenone or control for 4 h, then fixed and imaged to quantify numbers of yellow and red puncta. We observed a significant increase in the number of mitochondria and mitochondria within autophagosomes as well as mitochondria within autolysosomes in both untreated and Rotenone-treated BACE1 knocked-down cells compared to scrambled control (Figure 5D). Altogether, our observations of increased Parkin and PINK1 protein levels, Parkin translocation to mitochondria, and increased number of autolysosomes with mitochondrial cargo suggest a protective role of BACE1 on the mitochondria in the absence of which the mitophagic process is activated.

## 4. Discussion

In the present study, we attempted to establish the role of the BACE1 (and its homolog BACE2) in vitro system in the protection of epithelial cells against oxidative stress. Our results demonstrate that inhibition of BACE1 (but not BACE2) makes the RPE highly susceptible to oxidative stress resulting in reduced cell viability. BACE1 conferred a significant level of protection to mitochondria based on our observations that BACE1 inhibition reduces mitochondrial membrane potential, affects mitochondrial recycling, and, as a result, upregulates cellular apoptotic signaling. BACE1 knockdown resulted in a shift in mitochondrial reticular network dynamics towards greater mitochondrial fragmentation, which was associated with increased activity of the fission protein DRP1, while levels of fusion proteins such as MFN1, MFN2, and OPA1 are reduced. Overall our data show that BACE1 can modulate physiological mitochondrial dynamics, and its absence evokes greater alterations in these dynamics during oxidative stress. pSer637-DRP1 can be found in both the cytoplasm and the mitochondrial membrane, while at the mitochondrial membrane, it is reported to evoke mitochondrial fission via the PINK1/Parkin pathway [14], which is also what we observed. We believe that the pSer637/DRP1 ratio increase observed in our work is a negative feedback loop to remove the DRP1 that could be accumulated in the mitochondrial membrane. This is required because the presence of DRP1 on the mitochondrial outer membrane evokes mitochondria fission and cell death, and the phosphorylation at the 637 residue promotes its translocation back to the cytosol. The pSer616-DRP1 is more involved in mitochondrial fission during cell division. Increased mitochondrial fragmentation typically indicates cellular stress that can stimulate cell death pathways such as apoptosis [27]. While γ-secretase has been localized to mitochondria, active BACE1 has been predominantly shown to be associated with lipid rafts in the plasma membrane [18,19,28]. However, we now show that BACE1 colocalizes with mitochondria, supporting a role for BACE1 in preserving mitochondrial homeostasis. BACE1 knockdown activated pro-caspase 3, a key molecule in the intrinsic cellular apoptotic signaling, even when cells were not challenged by oxidative stressors, suggesting that loss of endogenous BACE1 primes the cell for mitochondrial membrane permeabilization and initiation of an apoptotic response. Previously, we and others have established the cytoprotective role of autophagy in the RPE that is activated under oxidative stress and removes damaged mitochondria [28,29,30]. As expected, oxidative stress significantly increased key molecules involved in mitophagy (PINK1 and Parkin) to segregate damaged mitochondria and remove them to prevent activation of cellular danger signals or apoptosis. BACE1 knockdown resulted in much higher levels of PINK1 and Parkin, suggesting that the damaged mitochondrial burden is greater in the absence of protection by BACE1. The dramatic increase of mitochondria within autophagosomes in BACE1 knocked down cells could be a combined outcome of autophagy induction and breakdown of autophagic flux. However, the increased mitochondrial translocation of Parkin, in addition to the higher number of mitochondrial cargos in autophagosomes and lysosomes of RPE cells, is consistent with increased mitophagy to remove dysfunctional mitochondria. Alterations in the mitophagic pathway by oxidative stress are similarly observed in other degenerative diseases involving BACE, such as Alzheimer’s disease and Parkinson’s disease [2,17,20].

Oxidative stress has been shown to activate BACE1 (including gene expression) in neuroblastoma cells and NT2 neurons [15,31] and that this is partly mediated by γ-secretase [32]. Elevated BACE1 expression is also reported in rat retinal plexiform layers when mitochondrial respiration is inhibited, resulting in increased cleavage of amyloid precursor protein, giving rise to cleaved product β-CTF as well as Aβ40 residues [33]. In agreement, we observed an increase in BACE1 activity under transient oxidative stress with a significant reduction on secreted Aβ40, suggesting that this type of Aβ is directly implicated in mitochondrial dysregulation in this model.

One consequence of mitochondrial damage and autophagic removal is the accumulation of the age pigment “lipofuscin” in post mitotic cells [34,35]. We have shown in a previous study that lipofuscin accumulation is significantly greater in RPE cells in vivo and in vitro following both pharmacological and knockdown approaches to inhibit BACE activity [5].

The mechanism by which BACE1 modulates the oxidative stress response and mitochondrial dynamics is unclear. However, γ-secretase complex, a key enzyme in the sequential cleavage of APP, is localized to mitochondria where it is reported to bind hypoxia inducible gene 1 and domain member 1A, is associated with metabolism, and binds to a mitochondrial serine protease [17,36,37]. Given the increasing number of substrates being identified for BACE1, it is likely that this too binds, and possibly cleaves, mitochondrial proteins, thus modulating their function. Further studies using proteomics and protein binding studies will be needed to answer this question.

Overall, BACE2 did not have a major effect on the susceptibility of cells to oxidative stress and only demonstrated a reduction in mitochondrial membrane potential in response to stress but did not significantly impact other mitochondrial readouts as observed when BACE1 was knocked down. We did not investigate the role of BACE2 further for two reasons: First, in our previous paper, we showed that only BACE1 knockout resulted in significant retinal pathology in the mouse, while BACE2 knockout did not result in significant retinal pathology, and the double BACE1/BACE2 knockout did not demonstrate any increase in retinal pathology in the mouse above that observed in BACE1 knockout alone [5]. Second, siRNA transfection with two different genes (e.g., BACE1 + BACE2) can result in greater uptake of one siRNA compared to the other, making data difficult to interpret.

## 5. Conclusions

In summary, we show that BACE1 (but not BACE2) confers protection against oxidative damage of mitochondria, a key organelle that is critical for cell survival and function. These data together suggest that BACE1 activity in neural tissues may, in addition to Aβ formation, play a significant role in mitochondrial maintenance and the autophagy pathway, both of which have been implicated in age-related neural degenerations such as Alzheimer’s disease and age-related macular degeneration [38,39].

## Data Availability

The data presented in this study are available in the article.

## Supplementary Materials

The following is available online at https://www.mdpi.com/article/10.3390/antiox10101539/s1, Figure S1: Uncropped Western blot images used in this study. Figure S2: BACE1 activity and RPE‐mitochondria association. ARPE19 cells BACE1 or VDAC protein expression were analyzed by Western blotting. Leftmost lanes were loaded with Biorad Precision Ladder. Molecular weights are in KDa units.
