# Supplementary material for: BACE1 Inhibition Increases Susceptibility to Oxidative Stress by Promoting Mitochondrial Damage
Source: Antioxidants (Basel). 2021 Sep 28;10(10):1539. doi: 10.3390/antiox10101539 (PMC8532805; doi:10.3390/antiox10101539)

Figure S1: Uncropped Western Blot images used in this study.

Supplementary Figure S1A. Confirmation of BACE1 and BACE2 in vitro. ARPE19 cells were transfected with scrambled non-specific siRNA or siRNAs targeted specifically towards BACE1 or BACE2 for 24 hours and the BACE1 and BACE2 protein expression was analyzed by Western blotting. Leftmost lanes were loaded with Biorad Precision Ladder. Molecular weights are in KDa units.

BACE1

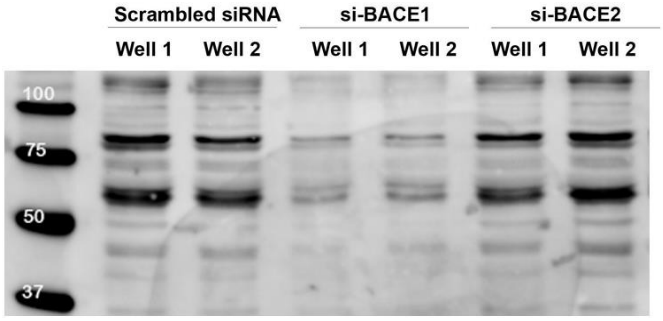

BACE2

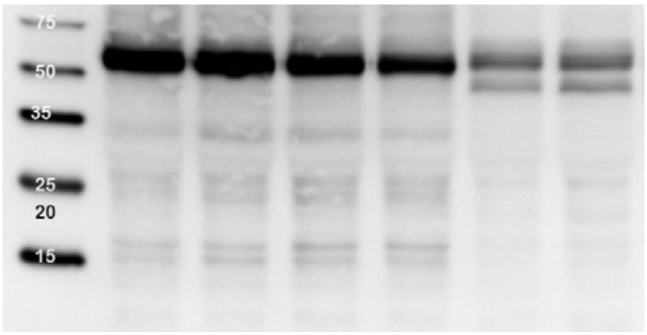

GAPDH

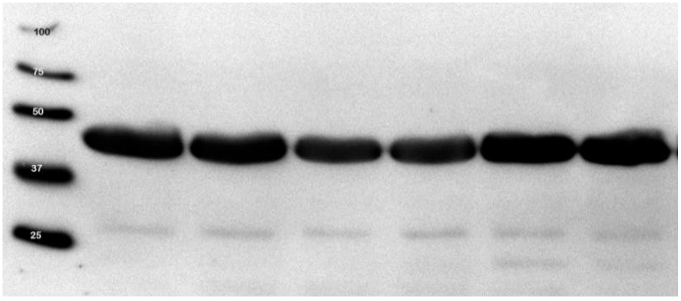

Supplementary Figure S1B. BAX and Caspase -3 protein analysis. ARPE19 cells were transfected with scrambled non-specific siRNA or siRNAs targeted specifically towards BACE1 or BACE2 for 24 hours and the caspase-3 and BAX protein expression were analyzed by Western blotting. Leftmost lanes were loaded with Biorad Precision Ladder. Molecular weights are in KDa units.

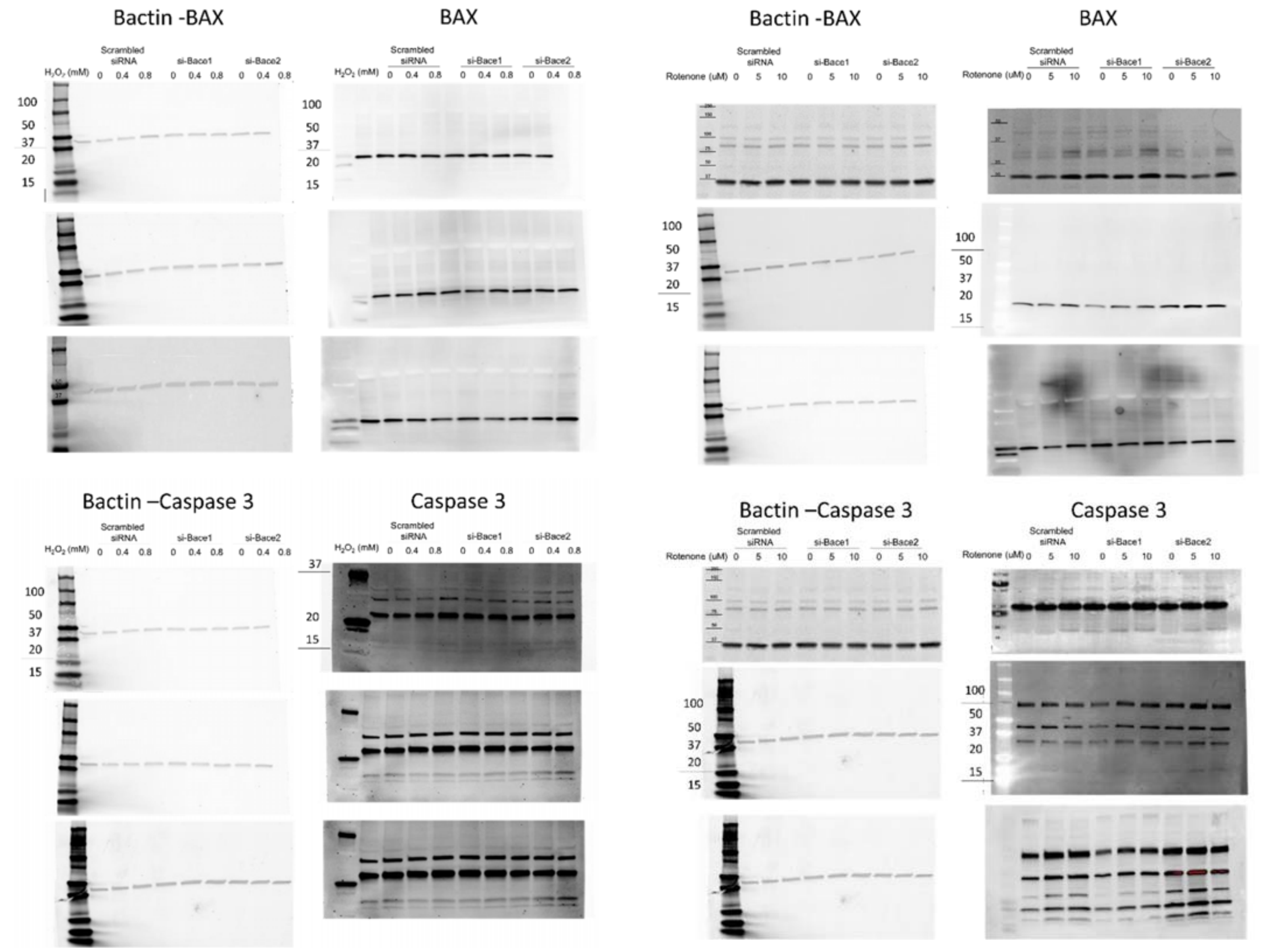

Supplementary Figure S1C. Mitochondrial fusion/fission proteins expression analysis. ARPE19 cells were transfected with scrambled non- specific siRNA or siRNAs targeted specifically towards BACE1 or BACE2 for 24 hours and the MFN1, MFN2, OPA1, pDRP1, and DRP1 protein expression were analyzed by Western blotting. Leftmost lanes were loaded with Biorad Precision Ladder. Molecular weights are in KDa units

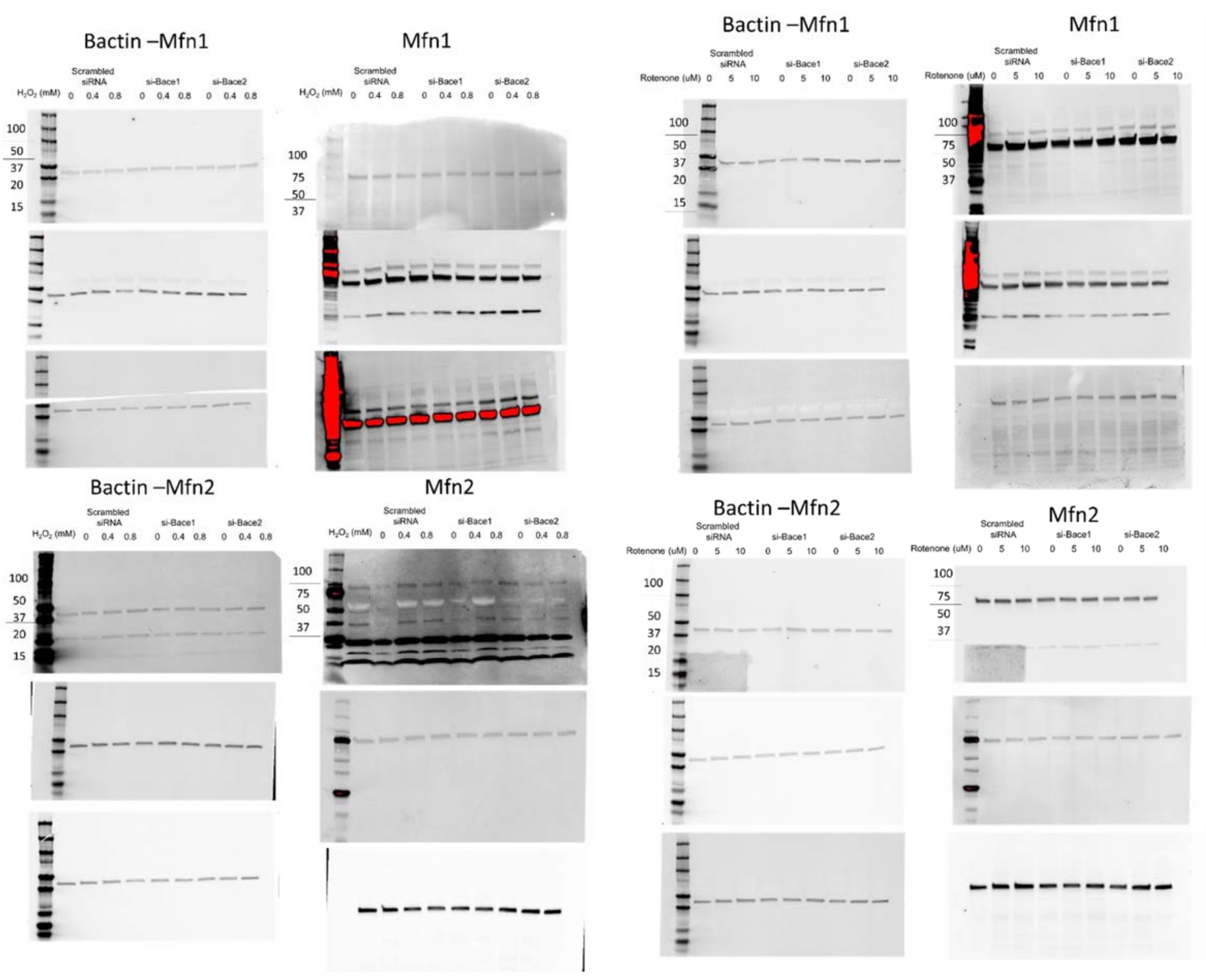

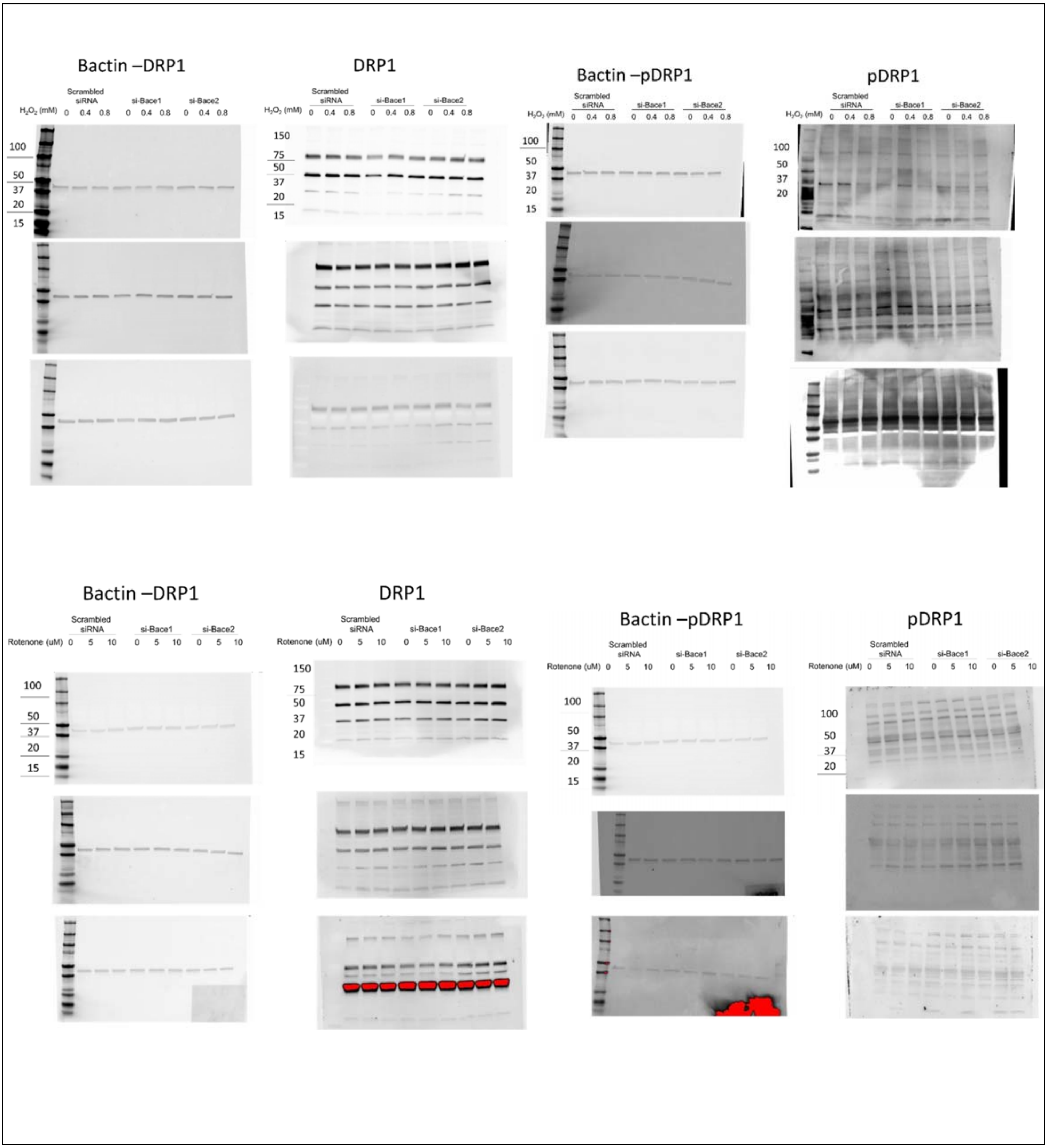
Bactin –DRP1
DRP1
Bactin –pDRP1
pDRP1
Scrambled siRNA
si-Bace1
si-Bace2
H2O2 (mM) 0 0.4 0.8 0 0.4 0.8 0 0.4 0.8
150
100
75
50
37
20
15
Rotenone (uM) 0 5 10 0 5 10 0 5 10

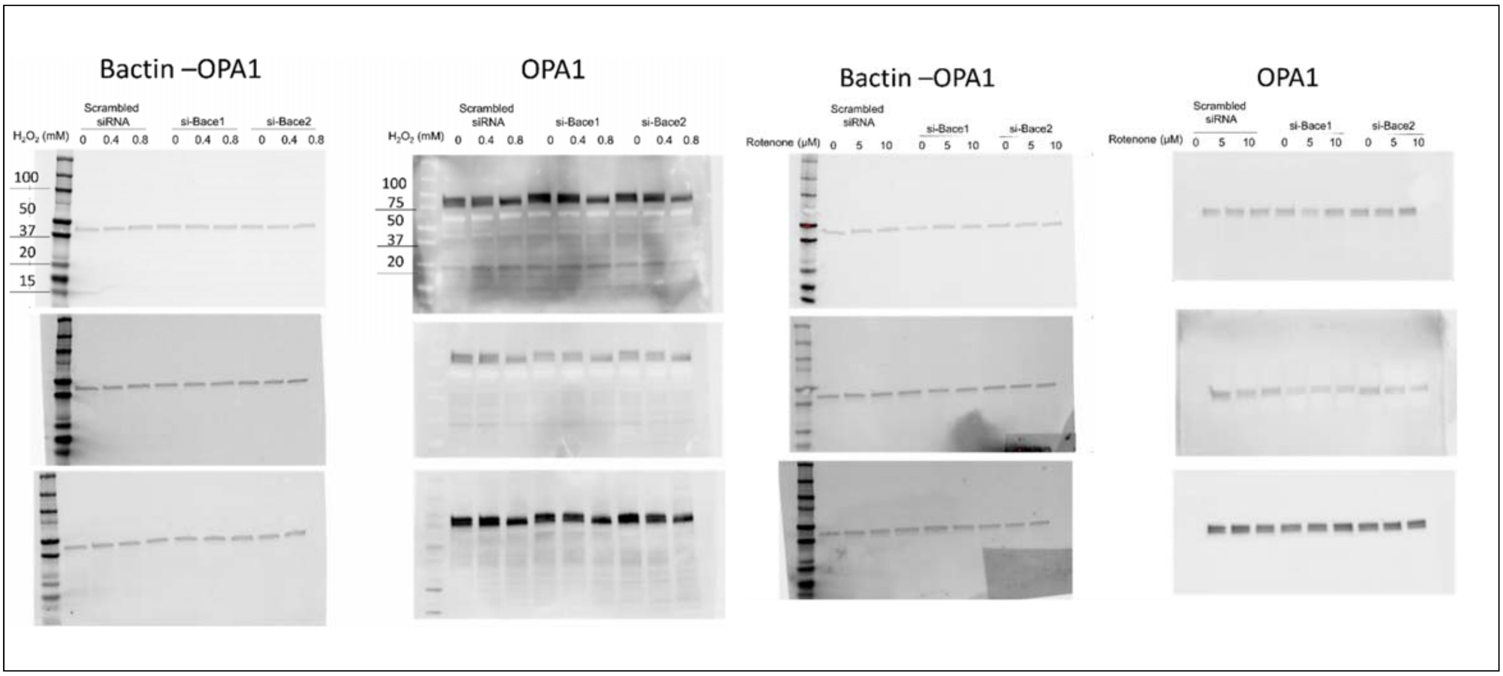
Bactin –OPA1
OPA1
Bactin –OPA1
OPA1
Scrambled siRNA
si-Bace1
si-Bace2
H_2O_2 (mM)
0 0.4 0.8
Rotenone (µM)
0 5 10
100
75
50
37
20
15

Supplementary Figure S1D. Mitochondrial fusion/fission proteins expression analysis. Proteins of interest were analyzed through chemoluminescent HRP detection and 3 to 4 times of exposure to image capture were applied in each sample. We show below some examples.

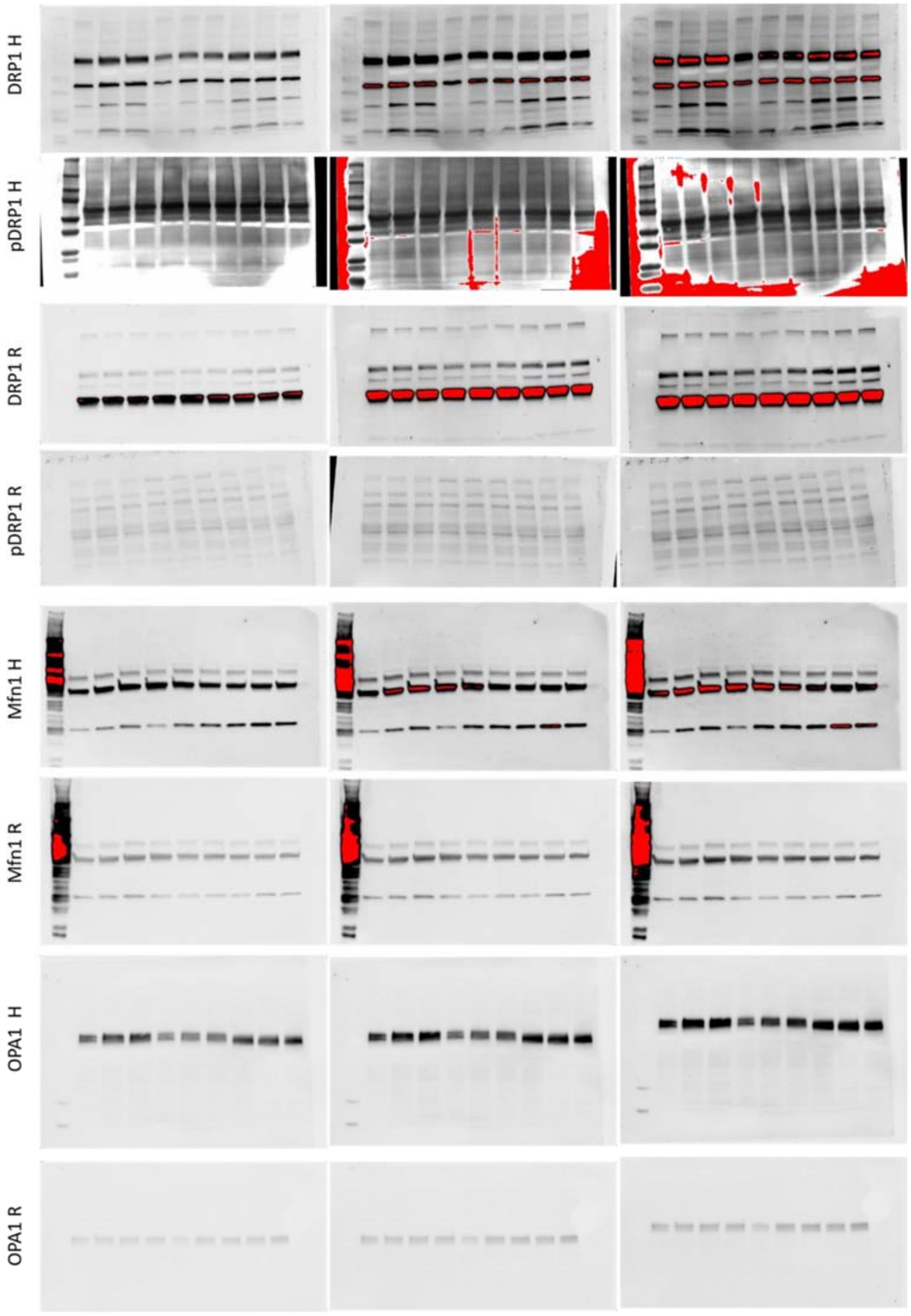

Supplementary Figure S1E. Autophagy and mitophagic machinery analysis in BACE1 and BACE2 KD ARPE19 cells under oxidative stress. ARPE19 cells were transfected with scrambled non-specific siRNA or siRNAs targeted specifically towards BACE1 or BACE2 for 24 hours and the PINK and Parkin protein expression were analyzed by Western blotting. Leftmost lanes were loaded with Biorad Precision Ladder. Molecular weights are in KDa units

PINK

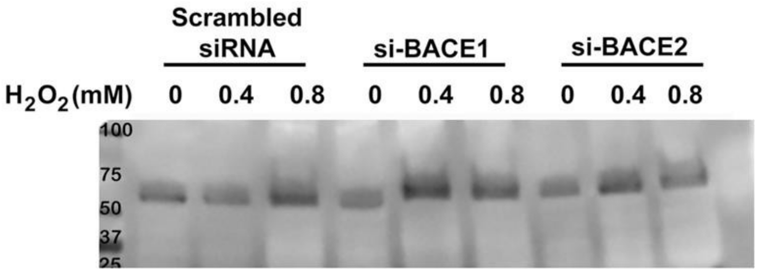

PARKIN

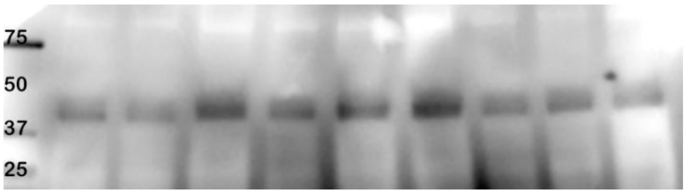

GAPDH

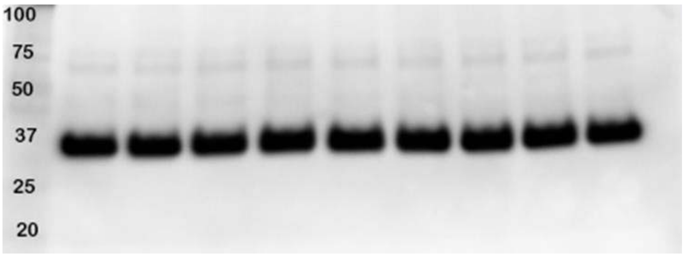

Supplementary Figure S2. BACE1 activity and RPE-mitochondria association. ARPE19 cells BACE1 or VDAC protein expression were analyzed by Western blotting. Leftmost lanes were loaded with Biorad Precision Ladder. Molecular weights are in KDa units.

BACE1

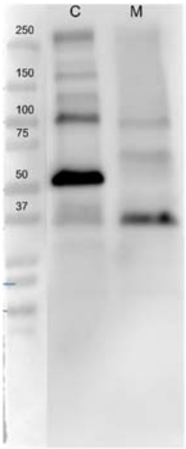

VDAC

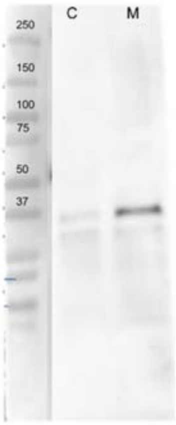

Supplement: Supplementary file 1 [file antioxidants-10-01539-s001.zip › antioxidants-1346665 - Supplementary .pdf]
